# Activation of *Saccharomyces cerevisiae* Mlh1-Pms1 Endonuclease in a Reconstituted Mismatch Repair System*

**DOI:** 10.1074/jbc.M115.662189

**Published:** 2015-07-13

**Authors:** Catherine E. Smith, Nikki Bowen, William J. Graham, Eva M. Goellner, Anjana Srivatsan, Richard D. Kolodner

**Affiliations:** From the ‡ Ludwig Institute for Cancer Research,; the § Department of Cellular and Molecular Medicine,; ¶ Moores-UCSD Cancer Center, and; the ‖ Institute of Genomic Medicine, University of California, San Diego School of Medicine, La Jolla, California 92093

**Keywords:** DNA recombination, DNA repair, DNA replication, mutagenesis, proliferating cell nuclear antigen (PCNA), replication factor C (RFC), yeast genetics, exonuclease 1, genome instability, Msh2-Msh6

## Abstract

Previous studies reported the reconstitution of an Mlh1-Pms1-independent 5′ nick-directed mismatch repair (MMR) reaction using *Saccharomyces cerevisiae* proteins. Here we describe the reconstitution of a mispair-dependent Mlh1-Pms1 endonuclease activation reaction requiring Msh2-Msh6 (or Msh2-Msh3), proliferating cell nuclear antigen (PCNA), and replication factor C (RFC) and a reconstituted Mlh1-Pms1-dependent 3′ nick-directed MMR reaction requiring Msh2-Msh6 (or Msh2-Msh3), exonuclease 1 (Exo1), replication protein A (RPA), RFC, PCNA, and DNA polymerase δ. Both reactions required Mg^2+^ and Mn^2+^ for optimal activity. The MMR reaction also required two reaction stages in which the first stage required incubation of Mlh1-Pms1 with substrate DNA, with or without Msh2-Msh6 (or Msh2-Msh3), PCNA, and RFC but did not require nicking of the substrate, followed by a second stage in which other proteins were added. Analysis of different mutant proteins demonstrated that both reactions required a functional Mlh1-Pms1 endonuclease active site, as well as mispair recognition and Mlh1-Pms1 recruitment by Msh2-Msh6 but not sliding clamp formation. Mutant Mlh1-Pms1 and PCNA proteins that were defective for Exo1-independent but not Exo1-dependent MMR *in vivo* were partially defective in the Mlh1-Pms1 endonuclease and MMR reactions, suggesting that both reactions reflect the activation of Mlh1-Pms1 seen in Exo1-independent MMR *in vivo*. The availability of this reconstituted MMR reaction should now make it possible to better study both Exo1-independent and Exo1-dependent MMR.

## Teaser

**Background:** Biochemical analysis of *S. cerevisiae* MMR mutants has been limited by a lack of reconstituted MMR reactions.

**Results:** 3′ nick-directed Mlh1-Pms1-dependent endonuclease and reconstituted MMR reactions were developed.

**Conclusion:** 3′ nick-directed MMR required the Mlh1-Pms1 endonuclease and was eliminated by mutations inactivating Exo1-independent MMR.

**Significance:** The reconstituted MMR reactions facilitated analysis of uncharacterized MMR mutants and the mechanism of Exo1-independent MMR.

## Introduction

DNA mismatch repair (MMR)^2^ plays a critical role in maintaining genome stability by excising nucleotides that are misincorporated as a result of DNA replication errors. Because MMR targets repair to the newly synthesized DNA strands, it reduces the frequency of mutations that occur as a result of these DNA replication errors (1–5). As a consequence, mismatch repair defects underlie the development of cancers associated with high rates of base substitution and frameshift mutations, with the latter often occurring in microsatellite sequences, resulting in a diagnostic phenotype called microsatellite instability (6–9). MMR also corrects mispaired bases in heteroduplex recombination intermediates, thus playing a role in gene conversion, and MMR helps to prevent recombination between divergent sequences preventing genome rearrangements formed by nonallelic homologous recombination (10–15). Whether MMR defects lead to genome rearrangements that play a role in the development of cancer is not clear, although MMR defective cancer cell lines have been described that have both microsatellite instability and increased genome rearrangements, suggesting that it does play a role (16).

The process of eukaryotic MMR initiates with the identification of mispairs in the genome by two different heterodimeric protein complexes that are homologs of the bacterial MutS dimer protein complex, the Msh2-Msh6 (MutS homolog) (sometimes called MutSα) and Msh2-Msh3 (sometimes called MutSβ) complexes (5, 17–22). These two complexes have distinct but overlapping mispair binding specificities and are partially redundant (5, 17, 18, 22–26). After mispair recognition, ATP binding by the Msh2-Msh6 and Msh2-Msh3 complexes induces a conformational change, which converts these complexes to a clamp form that slides along the DNA and licenses them to recruit a complex that is related to the bacterial MutL dimer protein complex (27–34). In *Saccharomyces cerevisiae*, the major MutL-related complex that functions in MMR is Mlh1-Pms1 (Mlh1-Pms2 in humans, sometimes called MutLα) (35, 36). Two other MutL-related complexes exist (37–39): the first, Mlh1-Mlh3, can substitute for Mlh1-Pms1 to a very limited extent (37, 39), and the second, Mlh1-Mlh2, has been proposed to be an accessory factor that acts in conjunction with Mlh1-Pms1 (40). Mlh1-Pms1 (and hMlh1-Pms2) has an Mn^2+^-dependent endonuclease activity that is activated *in vitro* on supercoiled circular DNA by the sliding clamp proliferating cell nuclear antigen (PCNA) and the clamp loader replication factor C (RFC) and an endonuclease activity that is activated on nicked, mispair-containing DNA by Msh2-Msh6 or Msh2-Msh3, PCNA, and RFC in the absence of added Mn^2+^ (41–45). This endonuclease nicks the strand containing a pre-existing nick, presumably to provide an entry site for a mispair excision reaction (4, 46). However, the role of additional nicks in the DNA is unclear given that pre-existing nicks, and in particular those that would occur at high density on the lagging DNA strand because of discontinuous DNA synthesis, can in principle provide an entry site for the mispair excision reaction.

Exonuclease 1 (Exo1) is thought to play a role in the excision step of MMR (47–51). However, in *S. cerevisiae*, deletion of *EXO1* causes only a minimal MMR defect, resulting in an increase in mutation rate ranging from 0.1 to 10% of the increase caused by complete loss of MMR (48, 52). Defects in murine Exo1 also cause only a partial loss of MMR (53), and similarly, mutations in human Exo1 have not been linked to the development of cancer (54). These results suggest that additional excision mechanisms must play a role in MMR. The proposed alternative excision mechanisms include strand displacement synthesis past a mispaired site by DNA polymerase δ (55), excision by the editing exonuclease activities of DNA polymerases (56), and a mechanism that might involve iterative nicking by the Mlh1-Pms1 endonuclease (44, 45). However, additional studies will be required to establish which if any of these mechanism(s) act as alternatives to Exo1-mediated excision.

Biochemical reconstitution studies have defined two types of mispair-directed excision/repair reactions. In one reaction, reconstituted with both *S. cerevisiae* and human proteins, a combination of Msh2-Msh6 or Msh2-Msh3, Exo1, DNA polymerase δ, the single-stranded DNA binding protein replication protein A (RPA), PCNA, and RFC can promote the repair of a circular mispaired substrate containing a nick on the 5′ side of the mispair (57–59). In this reaction, the mispair recognition factors and other proteins appear to stimulate excision by Exo1 past the mispair followed by repair DNA synthesis (50, 57). In a second reaction that has thus far only been reconstituted with human proteins, a combination of Msh2-Msh6, hMlh1-Pms2 (MutLα), Exo1, DNA polymerase δ, RPA, PCNA, and RFC can promote the repair of a circular mispaired substrate containing a nick on the 3′ side of the mispair (58). In this reaction, the hMlh1-Pms2 endonuclease is activated to generate nicks 5′ to the mispair, which then allows 5′ excision and subsequent gap filling to occur (41).

In comparison to human MMR, the genetics of *S. cerevisiae* MMR has been extensively characterized, but the lack of a complete range of reconstituted MMR systems has limited the ability to biochemically characterize the diversity of hypomorphic mutations available in different *S. cerevisiae* MMR genes. Here, we have reconstituted an Mlh1-Pms1 endonuclease-dependent MMR reaction *in vitro* using purified *S. cerevisiae* proteins. In addition, we used this system to study mutations that affect steps in the activation of the Mlh1-Pms1 endonuclease, including mutations that selectively inactivate Exo1-independent MMR (44, 45, 52, 60), to further characterize the role of the Mlh1-Pms1 endonuclease in facilitating MMR.

## Experimental Procedures

### 

#### 

##### Protein Purification

Mutations resulting in the Pms1-A99V or Pms1-G19D amino acid substitutions were introduced into the Pms1 expression vector pRDK1099 *LEU2 GAL10-PMS1-FLAG* by standard site-directed mutagenesis methods, and the expected sequences of the mutant *PMS1* genes were verified by DNA sequencing essentially as previously described (44). The methods for purifying Exo1, DNA polymerase δ, RPA, Msh2-Msh6, Msh2-Msh6-F337A, Msh2-Msh3, RFC-Δ1N, and PCNA have been described previously (57) and in some cases were adapted from methods described in other studies (24, 61–66); many of the preparations of these proteins used were those described previously (57). Mlh1-Pms1, Mlh1-Pms1-A99V, Mlh1-Pms1-G19D, Mlh1-Pms1-E707K, and Mlh1-Pms1-C848S were purified as described previously (33, 44), and many of the protein preparations used were those described previously (33, 44). Msh2-Msh6-FF33AA was the protein preparation described in Ref. 61, and Msh2-Msh6-S1036P and Msh2-Msh6-G1142D were the protein preparations described in Ref. 33.

##### Nick-directed Endonuclease Assays

Mlh1-Pms1 endonuclease assays were performed in 40-μl reactions containing 20 mm HEPES-KOH, pH 7.6, 140 mm KCl, 5 mm MgCl_2_, 0.5 mm MnSO_4_, 2 mm ATP, 1 mm DTT, 0.2 mg/ml BSA, 1.2% (w/v) glycerol, 195 fmol of Msh2-Msh6, 145 fmol of Mlh1-Pms1, 110 fmol of RFC-Δ1N, 145 fmol of PCNA (PCNA trimers), and 100 ng (52 fmol) of a pBS-SK-derived +1 (+T) mispaired plasmid substrate with a nick in the +T strand at the AflIII site constructed as previously described (57, 67). For one experiment, a homoduplex DNA containing an AT base pair with the A in the strand containing the nick at the AflIII site was constructed as previously described (57, 67). All of the reaction components including the substrate DNA were combined in a master mix followed by addition of the proteins and any reaction components that were varied in individual experiments. The reactions were incubated at 30 °C for 30 min and then terminated by addition of 30 μl of 0.35% SDS, 0.3 mg/ml proteinase K, 400 mm NaCl, 0.3 mg/ml glycogen, and 13 mm EDTA followed by incubation at 55 °C for 15 min. The DNA present in the samples was then purified by phenol extraction and ethanol precipitation and linearized by digestion with ScaI. The digestion products were then analyzed by electrophoresis through a 1% denaturing agarose gel followed by Southern blotting with probes specific for either the nicked or continuous strands of the substrate DNA (41).

##### Denaturing Agarose Gels

**FIGURE 1.**
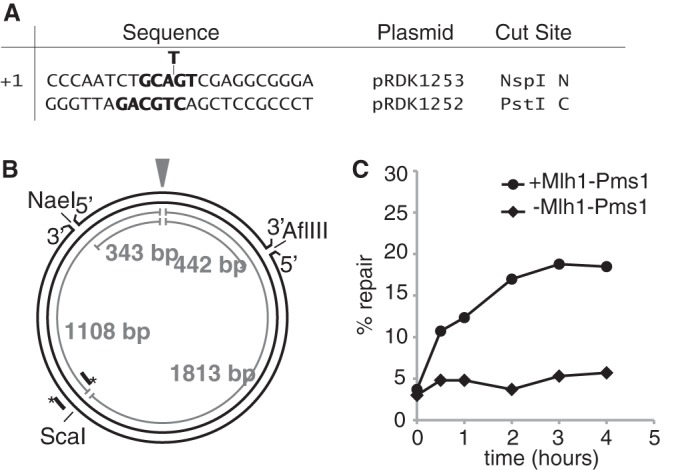
**Reconstitution of Mlh1-Pms1-dependent MMR of a +T insertion mispair-containing plasmid *in vitro*.** *A*, sequence of the polylinker region between the ApaI and BamHI sites of the +1 (+T) insertion mispaired substrate indicating the mispair, the restriction sites in each strand, and the plasmid from which each strand was derived. *N*, nicked strand; *C*, continuous strand. *B*, map of the complete pBluescript plasmid substrate showing the positions of the various features used in the assays presented and the relevant distances between key sites. The mispair is indicated by the *arrowhead*. The positions of the hybridization probes used to detect the N and C strands are shown by the *parallel lines* with an *asterisk* indicating the 5′ ends of the probes. *C*, time course of repair of the +1 (+T) substrates containing a 3′ nick at the AflIII site in reactions with or without Mlh1-Pms1 as indicated, for the indicated times. Repair was detected by digesting the product DNA with PstI and ScaI, fractionating the repair products by agarose gel electrophoresis and quantifying the repair-specific DNA species seen on the gels after staining with ethidium bromide. 100% repair is repair of 100 ng or 52 fmol of substrate.

To perform denaturing agarose gel electrophoresis (41), a melted 1.1% agarose gel was first made in distilled water. The liquid agarose was cooled to ∼55 °C; NaOH and EDTA were added to final concentrations of 50 and 2 mm, respectively, resulting in a final agarose concentration of 1%; and the solution was poured into an appropriate slab gel. Once solidified, the gels were equilibrated in running buffer consisting of 50 mm NaOH and 2 mm EDTA for 30 min. Ten nanograms of DNA from the endonuclease reaction was then combined with denaturing agarose gel loading dye consisting of 200 mm NaOH, 40 mm EDTA, 10% Ficoll (w/v) and 0.1% bromcresol blue and loaded onto the gel, which was then subjected to electrophoresis at 25 V for 3 h. Next, the gels were neutralized using a buffer consisting of 1.5 m NaCl and 0.5 m Tris, pH 7, for 30 min. DNA from the neutralized gels was then transferred to nylon membranes and probed with singly biotinylated probes for either the nicked (5′-attatcccgtattgacgccgggcaagagcaactcggtcgccgcatacact) or continuous strand (5′-agtgtatgcggcgaccgagttgctcttgcccggcgtcaatacgggataat), which hybridized to the substrate DNA adjacent to the ScaI site, on the NaeI side of the ScaI site following a previously published procedure (41) (see Fig. 1). Visualization of the Southern blots was performed using a Thermo Scientific chemiluminescent nucleic acid detection module, and the chemiluminescent signal was detected in the linear range using a Bio-Rad ChemiDoc MP imaging system. The percentage of nicked product DNA was calculated as the amount of nicked product DNA that was smaller than 1.55 kb (nicked strand) or 2.92 kb (continuous strand) divided by the combined signal of both the nicked and linear DNAs. In many experiments, the amount of nicked product observed was normalized to the amount of nicked product observed in the complete wild-type protein reaction. In these experiments, 100% nicking ranged from 4% (2 fmol) to 34% (18 fmol) of the substrate nicked (for an example of an individual experiment, see Figs. 3 and 7). The standard error was calculated from the results of three or more independent experiments and is indicated by the error bars in individual figures.

##### Reconstituted Mlh1-Pms1-dependent MMR Assays

Reactions were performed essentially as previously described (57) with the modification that the reactions were performed in two stages. In the first stage, 195 fmol of Msh2-Msh6, 145 fmol of Mlh1-Pms1, 110 fmol of RFC-Δ1N, and 145 fmol of PCNA were incubated for 10 min at 30 °C with 100 ng (52 fmol) of +T mispaired substrate with a strand discontinuity at the AflIII site (57) in a final volume of 5 μl. The proteins and DNA were combined in 2.5 μl and mixed with 2.5 μl of a master reaction buffer mix containing 33 mm Tris, pH 7.6, 75 mm KCl, 2.5 mm ATP, 1.66 mm glutathione, 8.3 mm MgCl_2_, 80 μg/ml BSA, 200 μm dNTPs, and 1 mm MnSO_4_. Following the 10-min incubation period at 30 °C, 195 fmol of Msh2-Msh6, 145 fmol of Mlh1-Pms1, 110 fmol of RFC-Δ1N, 145 fmol of PCNA (PCNA trimers), 40 fmol of DNA polymerase δ, 2.1 fmol of Exo1, and 900 fmol of RPA were added to the initial 5 μl along with H_2_O as required and 2.5 μl of a modified version of the above master reaction buffer mix lacking MnSO_4_ bringing the final reaction volume to 10 μl followed by a 2-h incubation period at 30 °C. The first stage reaction contained final concentrations of 0.5 mm MnSO_4_ and 4.2 mm MgCl_2_, and the second stage reaction contained final concentrations of 0.25 mm MnSO_4_ and 4.2 mm MgCl_2_; we did not add additional MnSO_4_ to the second stage reaction because the MnSO_4_ present in the first stage reaction was sufficient to support full activity. In reactions with Zn^2+^, ZnSO_4_ was substituted for MnSO_4_ at the same concentration as indicated above for MnSO_4_. Note that because KCl was present in the different protein dilutions, the final KCl concentration in the repair reaction was 100 mm. Repair was monitored by measuring the extent of restoration of a PstI site located at the site of the mispair by agarose gel electrophoresis as previously described. In many experiments, the amount of repair observed was normalized to the amount of repair observed in the complete reaction. In these experiments, 100% repair ranged from 7.7% (4.1 fmol) to 29% (15.3 fmol) of the substrate repaired (for an example of an individual experiment, see Fig. 1). The S.E. was calculated from the results of three or more independent experiments and is indicated by the error bars in individual figures.

## Results

### 

#### 

##### Mlh1-Pms1-dependent MMR Catalyzed by Purified S. cerevisiae Proteins

In a previous study, we demonstrated that a combination of six purified *S. cerevisiae* proteins including Msh2-Msh6 (or Msh2-Msh3), Exo1, RPA, DNA polymerase δ, PCNA, and RFC-Δ1N (or RFC) catalyzed mismatch-dependent repair of a mispaired substrate containing a nick at either an NaeI site 343 bp 5′ or at an AflIII site 442 bp 3′ (*i.e.* 2,479 bp 5′) from the mispair (57). Both repair reactions were mediated by 5′ → 3′ excision catalyzed by Exo1, and addition of the Mlh1-Pms1 endonuclease to either reaction had no effect on the efficiency or extent of repair (57). In the present study, we aimed to develop an Mlh1-Pms1 endonuclease-dependent MMR reaction using a mispaired substrate containing a +1 (+T) insertion mispair and a nick at the AflIII site 3′ to the mispair. To achieve this, three reaction parameters were investigated including: 1) reducing the amounts of Exo1 to prevent mispair-dependent 5′ → 3′ excision initiating at the AflIII site from reaching the mispair, which would mask any repair initiating from nicks introduced by Mlh1-Pms1; 2) separating the reaction into two reaction stages to allow for the interaction of Mlh1-Pms1 with substrate DNA; and 3) inclusion of Mn^2+^ in the reactions at the concentrations previously used to support the Mn^2+^-dependent nicking activity of Mlh1-Pms1 (or human Mlh1-Pms2) (41, 42, 44) in addition to Mg^2+^, to achieve optimal Mlh1-Pms1 endonuclease activation. This facilitated the development of an optimal reaction in which a 10-min first stage reaction containing Mlh1-Pms1, Msh2-Msh6, PCNA, RFC-Δ1N, Mn^2+^, Mg^2+^, ATP, and substrate DNA was then added to a second stage reaction mixture containing Msh2-Msh6, PCNA, RFC-Δ1N, Exo1, RPA, DNA polymerase δ, Mg^2+^, and ATP followed by incubation for different times. Mlh1-Pms1-dependent repair of the nicked strand of the substrate DNA occurred over a 2-h period and was detected by cleavage with PstI, whose recognition sequence in the discontinuous strand was restored by MMR at the mispair site and ScaI to produce a diagnostic pair of 1.1- and 1.8-kb fragments (Fig. 1).

**FIGURE 2.**
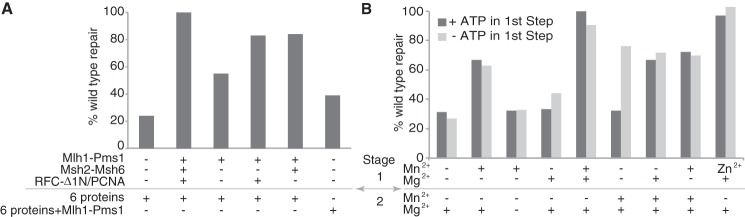
**Order of protein addition and divalent cation requirements for Mlh1-Pms1-dependent MMR *in vitro*.** Two-stage repair reactions containing the +1 (+T) substrate containing a 3′ nick at the AflIII site were performed for 2 h as described in the legend to Fig. 1 and under “Experimental Procedures.” In all cases, the level of repair obtained was normalized to the level of repair observed in the complete, standard repair reaction. *A*, the presence of individual proteins in the two reaction stages is indicated by the key below the histogram. *6 proteins* in the second stage indicates the presence of Msh2-Msh6, Exo1, PCNA, RFC-Δ1N, RPA, and DNA polymerase δ. *B*, the presence of MnSO_4_ (or ZnSO_4_) and/or MgCl_2_ in the two reaction stages at the final concentrations used in the different reaction stages under the standard reaction conditions (see “Experimental Procedures”) is indicated by the key below the histogram. ATP was present or absent in the first stage reaction as indicated; however, ATP was added to the second stage reaction in both cases.

To further evaluate the reaction conditions, the requirement of different proteins and divalent cations in the first stage reaction were examined (Fig. 2). The +T mispair was repaired to varying but significant levels when Mlh1-Pms1 alone, Mlh1-Pms1, and Msh2-Msh6 or Mlh1-Pms1, PCNA, and RFC-Δ1N were present in the first stage reaction, but the level of repair in each case was below that observed when Mlh1-Pms1, Msh2-Msh6, PCNA, and RFC-Δ1N were present in the first stage reaction (Fig. 2*A*). A lower but significant amount of repair above that seen in reactions without Mlh1-Pms1 was observed when no protein was present in the first stage reaction, and all seven proteins were present in the second stage reaction (Fig. 2*A*), indicating that the two-stage experimental design results in maximum levels of repair but is not absolutely required for Mlh1-Pms1-dependent repair to occur. Interestingly, the requirement for divalent cations in the first stage reaction depended on whether ATP was also present in the first stage reaction (Fig. 2*B*). In the presence of ATP in the first stage reaction, a divalent cation was also required in the first stage reaction; adding either Mn^2+^ or Mg^2+^ to first stage reactions containing ATP supported repair provided both Mn^2+^ and Mg^2+^ were ultimately present in the second stage reaction. In the absence of ATP in the first stage reaction, no divalent cation was required in the first stage, provided Mn^2+^, Mg^2+^, and ATP were present in the second stage reaction. Although significant levels of repair were observed when only Mg^2+^ (or Mn^2+^) was present in the repair reactions, the highest level of repair was observed in reactions that contained ATP, Mg^2+^, and Mn^2+^ in the first stage and additional Mg^2+^ in the second stage of the reaction, and therefore these conditions were used in the standard repair reaction in this study. We also found that Zn^2+^ would fully substitute for Mn^2+^ (Fig. 2*B*).

##### Efficient Activation of Nick-directed Mlh1-Pms1 Endonuclease Activity Requires Msh2-Msh6, RFC-Δ1N, PCNA, Mg^2+^, and Mn^2+^

**FIGURE 3.**
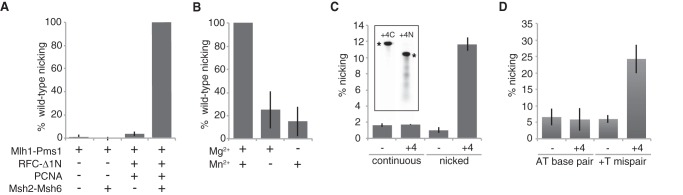
**Protein and divalent cation requirements for activation of nick-directed Mlh1-Pms1 endonuclease activity.** Mlh1-Pms1 endonuclease reactions containing the +1 (+T) substrate containing a 3′ nick at the AflIII site were performed for 30 min as described under “Experimental Procedures.” *A*, reactions containing 0.5 mm MnSO_4_ and 5 mm MgCl_2_ were performed with the combinations of Mlh1-Pms1, Msh2-Msh6, PCNA, and RFC-Δ1N indicated below the histogram, and nicking of the already nicked strand was monitored. In all cases, the level of nicking obtained was normalized to the level of nicking observed in the complete, standard reaction. *B*, reactions containing Mlh1-Pms1, Msh2-Msh6, PCNA, and RFC-Δ1N were performed with the combinations of 0.5 mm MnSO_4_ and/or 5 mm MgCl_2_ as indicated below the histogram, and nicking of the already nicked strand was monitored. In all cases, the level of nicking obtained was normalized to the level of nicking observed in the complete, standard reaction. *C*, reactions containing Mlh1-Pms1, Msh2-Msh6, PCNA, RFC-Δ1N, 0.5 mm MnSO_4_, and 5 mm MgCl_2_ were performed, and nicking of the already nicked strand and the continuous strand, as indicated below the histogram, was monitored. − and +*4* indicate the omission and inclusion of the four proteins, respectively. The percentage of substrate nicked is presented. The *inset* in *C* shows representative gels for the analysis of complete nicking reactions probed for the continuous strand (+*4C*) and the prenicked strand (+*4N*; 34% nicking) from an independent experiment using a different preparation of Mlh1-Pms1. The *asterisks* indicate the positions of the intact 2.92- and 1.55-kb fragments detected by the probes, respectively (also see Fig. 1*B* for additional details). *D*, reactions containing Mlh1-Pms1, Msh2-Msh6, PCNA, RFC-Δ1N, 0.5 mm MnSO_4_, 5 mm MgCl_2_, and either +T mispair or AT base pair homoduplex substrate DNA, as indicated below the histogram, were performed and nicking of the already nicked strand was monitored. − and +*4* indicate the omission and inclusion of the four proteins, respectively. The percentage of substrate nicked is presented.

To investigate the relationship between the Mlh1-Pms1-dependent repair reaction and the introduction of nicks by the Mlh1-Pms1 endonuclease, we developed an assay to measure endonucleolytic processing of the nicked strand of the AflIII mispaired substrate similar to assays described in previous studies (41, 42, 46). This endonuclease assay was similar to the first stage of the repair assay except that the reaction volume was 40 μl, the final salt concentration was 140 mm KCl, and the enzymatic activity was monitored by Southern blotting using a probe that hybridized to the nicked strand of the mispaired substrate adjacent to the unique ScaI site (Fig. 1). This allowed detection of smaller molecular weight species resulting from nicks on the 1.55-kb mispair-containing fragment between the ScaI and AflIII sites. Using this method, we observed the accumulation of up to 18 fmol of nicked product (Fig. 3*C*, *inset*) in reactions containing Mlh1-Pms1, Msh2-Msh6, PCNA, RFC-Δ1N, ATP, Mn^2+^, and Mg^2+^. Mn^2+^ was used at the concentration that was previously shown to support the Mn^2+^-dependent nicking activity of Mlh1-Pms1 (or human Mlh1-Pms2) (41, 42, 44). No smaller molecular weight species were observed when the reaction products were hybridized with a probe specific for the continuous strand of the substrate DNA, indicating that nicking only occurred on the strand containing the pre-existing nick (Fig. 3*C*). In addition, nicking of the already nicked strand was only observed above background when the substrate contained a mispair and was not observed when the substrate did not contain a mispair (Fig. 3*D*).

To further characterize the endonuclease activity, we tested the protein and divalent cation requirements for the activation of the Mlh1-Pms1 endonuclease. The endonuclease activity of Mlh1-Pms1 was completely dependent on the addition of Msh2-Msh6, RFC-Δ1N, and PCNA. Mlh1-Pms1 alone, Mlh1-Pms1 together with Msh2-Msh6, or Mlh1-Pms1 with RFC-Δ1N and PCNA did not did not have endonuclease activity (Fig. 3*A*). Msh2-Msh3, which was not previously tested for its ability to activate the Mlh1-Pms1 endonuclease but is known to activate the hMlh1-Pms2 endonuclease (43), was able to completely substitute for Msh2-Msh6 (see Fig. 6*A*). Finally, although a significant level of endonuclease activity was observed when either Mg^2+^ or Mn^2+^ alone was added to the reactions, higher levels of endonuclease activity were observed when both Mn^2+^ and Mg^2+^ were added to the reactions (Fig. 3*B*), and hence we included Mn^2+^ and Mg^2+^ in our standard reaction conditions.

##### Mlh1-Pms1 and PCNA Mutants That Are Defective in Exo1-independent MMR Have Defects in Activating the Mlh1-Pms1 Endonuclease and in Reconstituted MMR Reactions

To gain insights into the protein requirements and mechanisms of Mlh1-Pms1-dependent MMR *in vitro*, we investigated the effects of a series of mutations in different MMR genes on the ability of the respective MMR proteins to support MMR and activation of the Mlh1-Pms1 endonuclease *in vitro*. All of the experiments investigating MMR *in vitro* utilized the two-stage repair reaction conditions described above in which the first stage contained Msh2-Msh6, Mlh1-Pms1, PCNA, RFC-Δ1N, ATP, Mg^2+^, and Mn^2+^ and the second stage contained Msh2-Msh6, PCNA, RFC-Δ1N, Exo1, RPA, DNA polymerase δ, ATP, and Mg^2+^.

**FIGURE 4.**
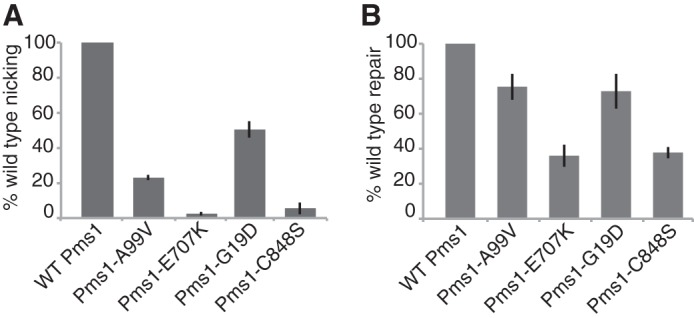
**Effects of *pms1* mutations that cause defects in the Mlh1-Pms1 active site and Exo1-independent MMR on activation of the Mlh1-Pms1 endonuclease and Mlh1-Pms1 dependent-MMR *in vitro*.** *A*, four-protein Mlh1-Pms1 endonuclease reactions with the +1 (+T) substrate containing a 3′ nick at the AflIII site, 0.5 mm MnSO_4_, and 5 mm MgCl_2_ were performed for 30 min as described in Fig. 3*A* and under “Experimental Procedures.” In all cases, the level of nicking obtained was normalized to the level of nicking observed in the complete, standard reaction. The presence of wild-type or mutant Mlh1-Pms1 complexes containing the indicated amino acid substitutions in the reactions is indicated by the key below the histogram. *B*, two-stage repair reactions with the +1 (+T) substrate containing a 3′ nick at the AflIII site were performed for 2 h as described in Fig. 1 and under “Experimental Procedures.” In all cases, the level of repair obtained was normalized to the level of repair observed in the complete, standard repair reaction. The presence of wild-type or mutant Mlh1-Pms1 complexes containing the indicated amino acid substitutions in the first stage reaction is indicated by the key below the histogram.

We initially investigated whether four different mutant Mlh1-Pms1 proteins could support MMR and Mlh1-Pms1 endonuclease activation *in vitro*. The Mlh1-Pms1-E707K and Mlh1-Pms1-C848S proteins that have amino acid substitutions that inactivate the endonuclease active site (44) were completely defective for endonuclease activation (Fig. 4*A*) and were as defective for repair as omitting Mlh1-Pms1 (Figs. 1*C* and 4*B*), indicating that the Mlh1-Pms1 endonuclease activity is required for MMR *in vitro*. We also tested the Mlh1-Pms1-A99V and Mlh1-G19D-Pms1 proteins, which have amino acid substitutions that cause defects in Exo1-independent MMR but not Exo1-dependent MMR (52). The Mlh1-Pms1-A99V and Mlh1-G19D-Pms1 proteins were partially defective for Mlh1-Pms1 endonuclease activation (Fig. 4*A*). Consistent with this result, substituting the Mlh1-Pms1-A99V and Mlh1-G19D-Pms1 proteins for Mlh1-Pms1 in the reconstituted MMR reaction resulted in modestly reduced repair but not to the extent observed in the endonuclease active site mutants (Fig. 4*B*); this difference likely reflects the fact that Mlh1-Pms1-A99V and Mlh1-G19D-Pms1 are only partially defective for endonuclease activation and given the longer incubation times used in the reconstituted MMR reactions likely contribute enough nicking to allow significant levels of repair to occur.

**FIGURE 5.**
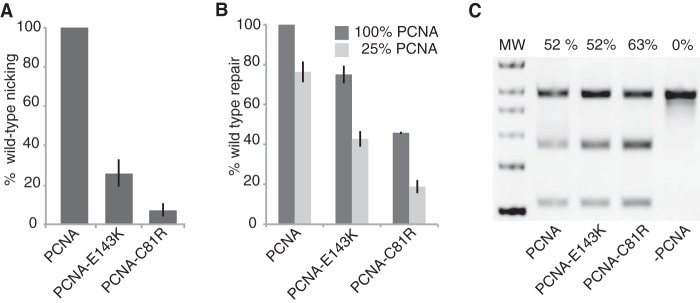
**Effects of *pol30* (PCNA) mutations that cause defects in Exo1-independent MMR on activation of the Mlh1-Pms1 endonuclease and Mlh1-Pms1-dependent MMR *in vitro*.** *A*, four-protein Mlh1-Pms1 endonuclease reactions with the +1 (+T) substrate containing a 3′ nick at the AflIII site, 0.5 mm MnSO_4_, and 5 mm MgCl_2_ were performed for 30 min as described in Fig. 3*A* and under “Experimental Procedures.” In all cases, the level of nicking obtained was normalized to the level of nicking observed in the complete, standard reaction. The presence of wild-type or mutant PCNAs containing the indicated amino acid substitutions in the reactions is indicated by the key below the histogram. *B*, two-stage repair reactions with the +1 (+T) substrate containing a 3′ nick at the AflIII site were performed for 2 h as described in Fig. 1 and under “Experimental Procedures.” In all cases, the level of repair obtained was normalized to the level of repair observed in the complete, standard repair reaction. The presence of wild-type or mutant PCNAs containing the indicated amino acid substitutions at either the normal concentration or 25% of the normal concentration is indicated by the key above the histogram. *C*, repair reactions with the +1 (+T) substrate containing a 5′ nick at the NaeI site were performed for 3 h exactly as previously described (57). The presence of wild-type or mutant PCNAs containing the indicated amino acid substitutions is indicated by the key below the gel image, and the percentage of repair is indicated above each lane. *MW*, molecular weight markers.

Because the mutant Mlh1-Pms1 proteins that have defects in Exo1-independent but not Exo1-dependent MMR had reduced activity in both mispair-directed Mlh1-Pms1 endonuclease activation and reconstituted MMR reactions, we next analyzed two mutant PCNA proteins, PCNA-E143K and PCNA-C81R. PCNA-E143K was originally identified in genetic screens for *pol30* mutations causing defects in Exo1-independent but not Exo1-dependent MMR (52), and PCNA-C81R was originally identified in a screen for *pol30* mutations causing general MMR defects (68). The *pol30-C81R* mutation was later found to cause much stronger defects in Exo1-independent MMR compared with Exo1-dependent MMR (45, 69). These two mutants were previously shown to have altered interactions with Msh2-Msh6 but to be fully proficient for supporting Mlh1-Pms1-mediated nicking of supercoiled homoduplex DNA substrates in reactions containing Mlh1-Pms1, RFC-Δ1N, and PCNA (45). The PCNA-E143K mutant was severely, but not completely, compromised in Mlh1-Pms1 endonuclease activation, whereas the PCNA-C81R mutant was almost completely defective in Mlh1-Pms1 endonuclease activation (Fig. 5*A*). Consistent with this observation, the PCNA-E143K mutant was partially defective in supporting MMR *in vitro*, whereas the PCNA-C81R showed an even greater defect in supporting MMR *in vitro*, but neither was completely defective for supporting MMR *in vitro*, even when the level of PCNA present in the repair reactions was reduced to 25% of the amount of PCNA present in the standard repair reaction (Fig. 5*B*). A control experiment showed that these mutant PCNA proteins were proficient for supporting repair of the 5′ nicked substrate whose repair does not require Mlh1-Pms1 (57), indicating that these mutant PCNA proteins are proficient in the PCNA-dependent gap filling reaction (Fig. 5*C*). This indicates that the PCNA-E143K and PCNA-C81R defects specifically reduced the ability of PCNA to support the activation of the Mlh1-Pms1 endonuclease on nicked mispaired substrates.

##### Mispair Recognition and Mlh1-Pms1 Recruitment but Not Sliding Clamp Formation or Interaction with PCNA by Msh2-Msh6 Are Required for Mlh1-Pms1 Endonuclease Activation and MMR in Vitro

**FIGURE 6.**
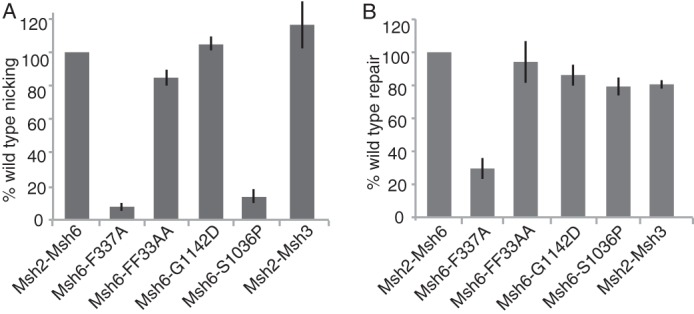
**Effect of mispair recognition, PCNA interaction, sliding clamp formation, and Mlh1-Pms1 recruitment-defective *msh6* mutations on activation of Mlh1-Pms1 endonuclease activity and Mlh1-Pms1-dependent MMR *in vitro*.** *A*, four-protein Mlh1-Pms1 endonuclease reactions with the +1 (+T) substrate containing a 3′ nick at the AflIII site, 0.5 mm MnSO_4_, and 5 mm MgCl_2_ were performed for 30 min as described in Fig. 3*A* and under “Experimental Procedures.” In all cases, the level of nicking obtained was normalized to the level of nicking observed in the complete, standard reaction. The presence of wild-type or mutant Msh2-Msh6 complexes containing the indicated amino acid substitutions in the reactions is indicated by the key below the histogram. *B*, two-stage repair reactions with the +1 (+T) substrate containing a 3′ nick at the AflIII site were performed for 2 h as described in Fig. 1 and under “Experimental Procedures.” In all cases, the level of repair obtained was normalized to the level of repair observed in the complete, standard repair reaction. The presence of wild-type or mutant Msh2-Msh6 complexes containing the indicated amino acid substitutions or Msh2-Msh3 in the reactions is indicated by the key below the histogram.

We next investigated a series of mutations affecting the Msh2-Msh6 complex for their effects on activation of Mlh1-Pms1 endonuclease activity and Mlh1-Pms1-dependent MMR by analyzing the following four mutant Msh2-Msh6 complexes: 1) the Msh2-Msh6-FF33AA mutant that is defective for interacting with PCNA through the Msh6 PIP Box motif (61, 70); 2) the Msh2-Msh6-F337A mutant that has defects in mispair recognition (19, 57); and 3) two dominant mutant proteins including Msh2-Msh6-S1036P that has a defect in binding ATP at the Msh2 ATP-binding site and that does not form sliding clamps or recruit Mlh1-Pms1 (31–33); and Msh2-Msh6-G1142D that can bind ATP at both ATP binding sites does not form sliding clamps but can still recruit Mlh1-Pms1. We also tested whether Msh2-Msh3 could substitutefor Msh2-Msh6. Omission of Msh2-Msh6 or substitution of Msh2-Msh6 with the mispair recognition defective Msh2-Msh6-F337A resulted in a loss of activation of the Mlh1-Pms1 endonuclease and a significant reduction of repair in the reconstituted Mlh1-Pms1-dependent MMR reaction (Fig. 6). In addition, consistent with results obtained with human Msh2-Msh6 (MutSα) (71, 72), eliminating the ability of Msh6 to interact with PCNA (Msh2-Msh6-FF33AA) did not reduce activation of the Mlh1-Pms1 endonuclease or cause a defect in MMR *in vitro* (Fig. 6). Msh2-Msh3 was able to substitute for Msh2-Msh6 in both the Mlh1-Pms1 endonuclease activation and the reconstituted Mlh1-Pms1-dependent MMR reactions consistent with previous analysis of the reconstituted 5′ nick-driven MMR reaction and studies on the activation of the hMlh1-Pms2 endonuclease (43, 59) (Fig. 6).

**FIGURE 7.**
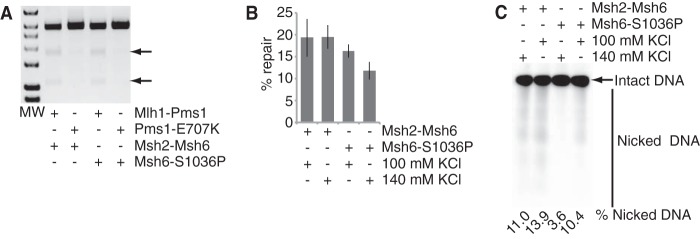
**The dominant mutant Msh2-Msh6-S1036P complex is a salt-sensitive mutant protein complex.** *A* and *B*, two-stage repair reactions with the +1 (+T) substrate containing a 3′ nick at the AflIII site were performed for 2 h as described in Fig. 1 and under “Experimental Procedures.” *A*, image of a gel showing the diagnostic pair of repair-specific 1.1- and 1.8-kb fragments indicated by the *arrows. MW* indicates molecular weight markers. The presence of either Msh2-Msh6 or Msh2-Msh6-S1036P and the presence of either Mlh1-Pms1 or Mlh1-Pms1-E707K is indicated by the key below the gel image. *B*, histogram indicating the percentage of total substrate repaired under the reaction conditions examined. The presence of either Msh2-Msh6 or Msh2-Msh6-S1036P and either 100 mm KCl or 140 mm KCl is indicated by the key below the histogram. *C*, Mlh1-Pms1 endonuclease reactions with the +1 (+T) substrate containing a 3′ nick at the AflIII site were performed for 30 min as described in Fig. 3 and under “Experimental Procedures.” An image of a gel showing the nicked products of the nicked strand is presented with the percentage of nicking indicated under each lane. The presence of either the normal KCl concentration (140 mm KCl) or the reduced KCl concentration (100 mm KCl) is indicated by the key above the gel image.

The two dominant Msh2-Msh6 mutants had distinctly different behaviors in the Mlh1-Pms1 endonuclease activation and reconstituted MMR reactions. The Msh2-Msh6-S1036P mutant was highly defective for activating the Mlh1-Pms1 endonuclease, whereas the Msh2-Msh6-G1142D was proficient for activating the Mlh1-Pms1 endonuclease, suggesting that Mlh1-Pms1 recruitment but not sliding clamp formation is required for activation of the Mlh1-Pms1 endonuclease (Fig. 6*A*). The Msh2-Msh6-S1036P and Msh2-Msh6-G1142D complexes both fully supported MMR *in vitro* (Fig. 6*B*), which in the case of the Msh2-Msh6-S1036P mutant was surprising because this mutant was highly defective in activating the Mlh1-Pms1 endonuclease. We confirmed that the reconstituted MMR reaction containing the Msh2-Msh6-S1036P complex required the Mlh1-Pms1 endonuclease and was significantly reduced when the endonuclease active site mutant Mlh1-Pms1-E707K was substituted for wild-type Mlh1-Pms1 (Fig. 7*A*). After examining the differences between the Mlh1-Pms1 activation assay and the *in vitro* MMR assay, we found that increasing the KCl concentration in the reconstituted MMR reaction from 100 to 140 mm, which is the KCl concentration in the Mlh1-Pms1 endonuclease activation assays, reduced the amount of repair in the presence of Msh2-Msh6-S1036P but not wild-type Msh2-Msh6 (Fig. 7*B*). Additionally, lowering the KCl concentration in the Mlh1-Pms1 endonuclease activation assay from 140 to 100 mm restored activation of the Mlh1-Pms1 endonuclease in the presence of the Msh2-Msh6-S1036P protein (Fig. 7*C*). This indicates that Msh2-Msh6-S1036P has a salt-sensitive defect in the activation of the Mlh1-Pms1 endonuclease.

## Discussion

A key challenge in elucidating MMR mechanisms is linking the biochemical properties of purified MMR proteins and reconstituted MMR reactions to MMR mechanisms *in vivo*. Achieving this would allow determining whether the biochemical properties of MMR proteins and reconstituted MMR reactions can account for MMR *in vivo* and allow identification of features of MMR that have not yet been reconstituted *in vitro*. To facilitate these efforts, in the present study we have reconstituted Mlh1-Pms1-dependent 3′ nick-directed MMR reactions, as well as Msh2-Msh6-dependent, Mlh1-Pms1 endonuclease reactions using *S. cerevisiae* proteins. Critical to this effort have been the development of a two-stage MMR reaction and the identification of an essential divalent cation requirement for MMR *in vitro*. The availability of this reconstituted MMR reaction now makes it possible to exploit the wealth of MMR-defective mutations identified in genetic studies to explore the relationships between the biochemical properties of MMR proteins and MMR mechanisms *in vivo*.

We observed that a mixture of Msh2-Msh6 (or Msh2-Msh3), Mlh1-Pms1, Exo1, RPA, PCNA, RFC, DNA polymerase δ, ATP, Mg^2+^, and Mn^2+^ repaired a mispaired plasmid substrate containing a +1 (+T) mispair and a 3′ nick located 442 bp from the mispair in an Mlh1-Pms1-dependent, Msh2-Msh6 mispair recognition-dependent reaction. We also observed a mispair-dependent, mispair-recognition-dependent, nick-directed nicking reaction catalyzed by Msh2-Msh6 (or Msh2-Msh3), Mlh1-Pms1, PCNA, and RFC under essentially the same reaction conditions. Three modifications of our previously reported reconstituted 5′ nick-directed MMR system were required to observe Mlh1-Pms1-dependent MMR *in vitro* (57). First, it was necessary to reduce the levels of Exo1 to limit long-patch 5′ nick-directed MMR from the AflIII nick that potentially obscures Mlh1-Pms1-dependent repair; however, it should be noted that this modification possibly reduces the efficiency of excision from nicks introduced by Mlh1-Pms1. Second, it was necessary to add both Mg^2+^ and Mn^2+^ to the reactions to observe optimal Mlh1-Pms1-dependent nicking and Mlh1-Pms1-dependent MMR *in vitro*. This is in contrast to previous studies of MMR reconstituted with human proteins and mispair-dependent nicking of DNA catalyzed by human Msh2-Msh6 (or Msh2-Msh3), Mlh1-Pms2 (MutLα), PCNA, and RFC or *S. cerevisiae* Msh2-Msh6, Mlh1-Pms1, PCNA, and RFC, which only required Mg^2+^; however, these studies did not appear to test whether addition of Mn^2+^ or Zn^2+^ would stimulate the reactions reported (41, 42, 46, 58, 59). The most likely explanation for this is that the divalent cation bound at the Mlh1-Pms1 endonuclease active site was lost during our purification of Mlh1-Pms1, creating a requirement for Mn^2+^ or Zn^2+^, because this metal binding site is not expected to bind Mg^2+^ (41, 44, 73, 74). Finally, it was necessary to develop a two-stage reaction where at a minimum, the first stage reaction contained Mlh1-Pms1 and substrate DNA, and the second stage reaction additionally contained all of the remaining reaction components. The endonuclease activity of Mlh1-Pms1 was required in the reconstituted MMR reaction and a combination of Msh2-Msh6 (or Msh2-Msh3), PCNA, and RFC was required to activate the Mlh1-Pms1 endonuclease to nick the mispaired substrate on the nicked strand under the reaction conditions used. The most likely mechanism of MMR under these conditions involves nicking of the substrate on the nicked strand close enough on the 5′ side of the mispair for the previously characterized short patch repair reaction to excise and resynthesize the nicked DNA strand in the vicinity of the mispair as previously shown for human MMR (41).

Optimal Mlh1-Pms1-dependent MMR required a first stage reaction containing Msh2-Msh6 (or Msh2-Msh3), Mlh1-Pms1, PCNA, RFC and substrate DNA, whereas inclusion of only Mlh1-Pms1 and substrate DNA in the first stage reaction resulted in ∼50% (Fig. 2*A*) of maximal activity. The inclusion of ATP alone did not support a functional first stage reaction, whereas the inclusion of ATP plus a divalent cation or divalent cations alone supported functional first stage reactions. Because the Mlh1-Pms1 endonuclease was inactive under several of the functional first stage reaction conditions (Mlh1-Pms1 alone with substrate DNA under any condition; Msh2-Msh6, Mlh1-Pms1, PCNA, RFC, and substrate DNA without ATP and divalent cations including Mn^2+^), the first stage reaction does not likely involve nicking of the substrate DNA but rather likely provides an opportunity for Mlh1-Pms1 to interact with the substrate DNA.

The MutL N-terminal domains and the equivalent Mlh1 and Pms1 N-terminal domains are known to associate on binding ATP, resulting in a ring-like conformation in which the associated N-terminal domains are joined to the dimerized C-terminal domains of MutL or Mlh1 and Pms1, respectively, by two unstructured linkers (73, 75–80). The N-terminal domains then dissociate from each other upon hydrolyzing ATP, which requires a divalent cation, and ADP release (76–79). Interestingly, the only conditions where the first stage reaction is functional are when the ATP binding and hydrolysis-driven Mlh1 and Pms1 N-terminal domain association and dissociation cycle is active (ATP and Mg^2+^ or Mn^2+^) or when the N-terminal domains of Mlh1 and Pms1 are not able to interact with each other (−ATP). In contrast, conditions that support stable association of the N-terminal domains of Mlh1 and Pms1 (ATP without Mg^2+^ or Mn^2+^) did not support a functional first stage reaction. This suggests that either the free N-terminal domains of Mlh1 and Pms1 or a region of one or both of the Mlh1 and Pms1 linkers that is blocked by the associated N-terminal domains of Mlh1 and Pms1 is critical for the activity of Mlh1-Pms1 in the first stage reaction.

Consistent with previously published studies (41, 42, 46), a combination of Msh2-Msh6 (or Msh2-Msh3), Mlh1-Pms1, PCNA, and RFC was found to promote nicking of the nicked strand of the mispaired substrate under essentially the same reaction conditions as the complete MMR reaction, provided the reactions contained ATP, Mg^2+^, and Mn^2+^. Analysis of different mutant proteins revealed that activation of the Mlh1-Pms1 endonuclease required mispair recognition by Msh2-Msh6 and key amino acid residues of the Mlh1-Pms1 endonuclease active site. However, consistent with previous studies of hMsh2-Msh6 (71), activation did not require the interaction between PCNA and the Msh6 PIP Box, which is consistent with a specific role of the PCNA-Msh6 PIP Box interaction in coupling MMR proteins to DNA replication (61, 69). Analysis of two dominant MMR-defective Msh2-Msh6 mutants (81) showed that the ability to promote mispair-dependent recruitment of Mlh1-Pms1 was important for activation of the Mlh1-Pms1 endonuclease but that the formation of ATP-induced mispair-dependent sliding clamps was not required (31–33). Overall, these results suggest that a mispair recognition-dependent ATP-induced conformational change in Msh2-Msh6, most likely needed for recruitment of Mlh1-Pms1 but not for sliding clamp formation, is required for activation of the Mlh1-Pms1 endonuclease, similar to the activation of the MutH endonuclease by MutS and MutL (82, 83). These results also suggest that formation of Msh2-Msh6 sliding clamps (27, 28, 30), which is important for MMR (31–33), likely plays a yet unknown role in MMR that is independent of recruitment and activation of the Mlh1-Pms1 endonuclease.

To investigate the relationship between activation of the Mlh1-Pms1 endonuclease in the four-protein system to the Exo1-independent and Exo1-dependent MMR pathways that have been identified in genetic studies, we investigated four mutant proteins that are defective in Exo1-independent MMR but are proficient in Exo1-dependent MMR (44, 45, 52). Two mutant PCNA proteins, PCNA-E143K and PCNA-C81R, which have altered interactions with Msh2-Msh6 but are fully proficient in PCNA- and RFC-dependent activation of the Mlh1-Pms1 endonuclease on supercoiled DNA substrates (45), were significantly but not completely defective in activating the Mlh1-Pms1 endonuclease on nicked mispaired substrates in the presence of Msh2-Msh6 and RFC. These results raise the possibility that some type of interaction between Msh2-Msh6 and PCNA is important for the activation of the Mlh1-Pms1 endonuclease, even though the interaction between the Msh6 PIP Box and PCNA is not required for activation of the Mlh1-Pms1 endonuclease. Similarly, two mutant Mlh1-Pms1 complexes having amino acid substitutions in the N-terminal domains of either Mlh1 or Pms1 that only compromise Exo1-independent MMR (52) were significantly but not completely defective in activation of the Mlh1-Pms1 endonuclease in the four-protein nicking reaction. Little is known about the specific biochemical defects caused by these two amino acid substitutions, although because of their locations (52) they could affect interactions between ATP and the N-terminal domains of Mlh1 and Pms1 (78, 79). Overall, these results support the hypothesis that higher levels of activation of the Mlh1-Pms1 endonuclease are required for Exo1-independent MMR compared with the levels of Mlh1-Pms1 endonuclease activation required to support Exo1-dependent MMR (44, 45).

The biochemical properties of the reconstituted Mlh1-Pms1-dependent MMR reaction generally corresponded to those of the Msh2-Msh6 (or Msh2-Msh3), PCNA, RFC, and mispair-dependent activation of the Mlh1-Pms1 endonuclease. These include the defects caused by the Msh6 mispair recognition-defective mutation, the Mlh1-Pms1 endonuclease active site mutations, the Exo1-independent MMR-specific mutations affecting PCNA and Mlh1-Pms1, and the defect caused by the Msh6-S1036P amino acid substitution at high KCl concentrations, as well as the lack of an effect of the Msh6 PIP Box-PCNA interaction-defective mutation and the Msh6-G1142D defect. Overall, these results suggest that the reconstituted Mlh1-Pms1-dependent MMR reaction corresponds to a reaction in which Mlh1-Pms1 endonuclease activation, potentially at the higher levels corresponding to that required for Exo1-independent MMR (44, 45), is coupled to a 5′ mispair-dependent excision reaction requiring Exo1 *in vitro* (50, 57, 84).

## Author Contributions

C. E. S. performed experiments on the four protein nicking reactions and revised the paper. N. B. performed experiments on reconstitution of mismatch repair reactions and revised the paper. W. J. G. performed experiments on the four protein nicking reactions and revised the paper. E. M. G. provided purified proteins and revised the paper. A. S. provided purified proteins and revised the paper. R. D. K. designed and coordinated the study and wrote and revised the paper.

## Footnotes

2 The abbreviations used are:

MMR: mismatch repair
PCNA: proliferating cell nuclear antigen
RFC: replication factor C
RPA: replication protein A
Exo1: exonuclease 1.
